# Other PHAC publications

**DOI:** 10.24095/hpcdp.46.4.05

**Published:** 2026-04

**Researchers from the Public Health Agency of Canada also contribute to work published in other journals and books. Look for the following articles published in 2025 and 2026:**

**Benkhalti Jandu M, Salzman T, Rahman P, Hashi A, Boland L, Drysdale M.** Piloting the novel Evidence Synthesis Equity Companion (ESEC) tool. Ann Epidemiol. 2025;112:94-101. https://doi.org/10.1016/j.annepidem.2025.11.002

Bioku AA, Lu B, Harris P, Sarimiye FO, Jimeta-Tuko J, Manoj N, **Olagunju TO**, et al. Coping strategies and perception of patients with breast cancer post-surgery in an underserved region of Nigeria. Psychooncology. 2026;35(1):e70370. https://doi.org/10.1002/pon.70370

Boucher LM, Lau J, **Scott MM**, Everett K, Gomes T, Tanuseputro P, et al. Relationship between palliative care and location of death among people with opioid use disorder: a retrospective cohort study. J Palliat Care. 2025:08258597251392965. https://doi.org/10.1177/08258597251392965

**Crompton L, Chu N, Pollock NJ**, Fluke J, **Tonmyr L**. Measuring social and community services for children, youth, and families in contact with the child welfare system: a scoping review. Child Prot Pract. 2026;8:100266. https://doi.org/10.1016/j.chipro.2025.100266

Hutt-Taylor K, Chamberland-Fontaine S, Bardekjian AC, Khan A, Duncan A, Mokdad A, […] **Prince SA**, et al. Improving cross-sectoral collaboration towards urban nature-based solutions: insights from a participatory workshop. Facets. 2025;10. https://doi.org/10.1139/facets-2025-0035

**Liu L, Contreras G, Thompson W**. Suicidal ideation and suicide attempts among adolescents in Canada based on the 2023 Canadian Health Survey on Children and Youth. Discover Public Health. 2025;22(1):826. https://doi.org/10.1186/s12982-025-01179-0

Pich-Renaud P, Buchan CA, Burton C, Chapdelaine H, Jeewa A, Morris SK, […] **Salvadori MI**, et al. Revaccination of individuals with cardiac adverse events following COVID-19 vaccination: a Canadian Immunization Research Network study. Vaccine. 2026;70:128016. https://doi.org/10.1016/j.vaccine.2025.128016

**Prince SA, Shan Y, Butler GP, Lang JJ**, McCormack GR, Colley RC. Comparing perceived and objective measures of neighbourhood built environments among youth and adults in Canada. Public Health. 2026;251:106110. https://doi.org/10.1016/j.puhe.2025.106110

**Pugh A, Dave S, Ebrahim M, Laroche JA**. Factors associated with COVID-19 non-vaccination among children and adolescents with chronic health conditions in Canada: a national cross-sectional study. Vaccine X. 2025;27:100753. https://doi.org/10.1016/j.jvacx.2025.100753

Sette S, Coplan RJ, **Ooi LL**, Zuffian A, Xiao B, Wong QJJ, et al. Measurement invariance of the Straightforwardly-Worded Social Interaction Anxiety Scale and associations with life satisfaction among emerging adults attending university in 10 countries. J Anxiety Disord. 2025;116:103092. https://doi.org/10.1016/j.janxdis.2025.103092

**Sharma P, Pollock NJ, Hovdestad W, Williams G, Tonmyr L**. Prevalence of out-of-home care among school-aged children in Canada, 2002–2018: an analysis of nationally-representative student survey data. Int J Public Health. 2025;70:1608481. https://doi.org/10.3389/ijph.2025.1608481

Shereefdeen H, **Thaivalappil A**, Young I, MacKay M. A pilot study on generative artificial intelligence’s reliability in qualitative research quality appraisal using CASP and JBI checklists. Inquiry. 2025;62. https://doi.org/10.1177/00469580251399374

